# Polymorphisms and Haplotypes in Candidate Genes Related to Angiogenesis and Endothelial Dysfunction in Preeclampsia

**DOI:** 10.1155/2012/914704

**Published:** 2012-04-05

**Authors:** Marcelo Rizzatti Luizon, Ana Carolina Taveiros Palei, Valeria Cristina Sandrim

**Affiliations:** ^1^Department of Pharmacology, Faculty of Medicine of Ribeirao Preto, University of Sao Paulo, Avenida Bandeirantes, 3900, 14049-900 Ribeirao Preto, SP, Brazil; ^2^Department of Pharmacology, Faculty of Medical Sciences, State University of Campinas, 13081-971 Campinas, SP, Brazil; ^3^Núcleo de Pós-Graduação e Pesquisa, Santa Casa de Belo Horizonte, Rua Domingos Vieira 590, 30150-240 Belo Horizonte, MG, Brazil

## Abstract

Valenzuela and colleagues have recently reviewed some polymorphisms in important candidate genes involved in different pathogenic mechanisms related to preeclampsia (PE) and concluded that various studies in different populations have identified maternal polymorphisms associated with PE. However, we would like to contribute to some studies regarding candidate genes related to angiogenesis and endothelial dysfunction in PE performed in the Brazilian population. Specifically, genotypes and haplotypes formed by polymorphisms of *VEGF*, *eNOS* and *MMP-9*, along with an example of the interaction among these genes in the prediction of PE. Our suggestions may provide additional information with clinical relevance to PE susceptibility.

Valenzuela et al. [1] have recently published an interesting *Review Article* in a special issue of *Journal of Pregnancy* discussing some polymorphisms in important candidate genes involved in different pathogenic mechanisms related to preeclampsia (PE). They have concluded that various studies in different populations have identified maternal polymorphisms associated with PE through candidate gene approaches [1]. However, some important references from studies performed in the Brazilian population were not cited, more specifically regarding the mentioned candidate genes related to *vascular and endothelial function.*

For example, our group has recently demonstrated a main effect of vascular endothelial growth factor (VEGF) genotypes and haplotypes involving three clinically relevant single nucleotide polymorphisms (SNPs) localized in the promoter region of *VEGF*; −2578C/A (rs699947), −1154G/A (rs1570360), and −634G/C (rs2010963) in the development of PE, but not with gestational hypertension (GH) [2]. When white and nonwhite pregnant women were considered together, no significant differences were found in the distributions of *VEGF* genotypes or haplotypes (*P* > 0.05). However, significant differences were found in genotypes distributions for two *VEGF* polymorphisms (−2578C/A and −634G/C; both *P* < 0.05) between the healthy pregnant (HP) and the PE groups when only white subjects were considered in the analysis [2]. Importantly, the haplotype including the alleles −2578C, −1154G, and −634C, which is associated with higher VEGF gene expression elsewhere [3], was less common in the PE group compared with the HP group (*P* = 0.0047 [2]). Moreover, we have previously reported marked interethnic differences in the distribution of these *VEGF* genotypes and haplotypes [4]. These differences could explain why we have found significant associations between *VEGF* genotypes and one *VEGF* haplotype with preeclampsia when only white women were considered in the analysis [2].

Regarding the endothelial nitric oxide synthase (eNOS), our group had previously examined the association of three clinically relevant polymorphisms in the promoter region (−786T/C, rs2070744), in intron 4 (a variable number of tandem repeats, VNTR) and in exon 7 (Glu298Asp, rs1799983) of *eNOS* with PE and GH [5]. No differences were observed in the frequencies of genotypes and alleles of the three polymorphisms among PE, GH, and HP groups (all *P* > 0.05). However, the haplotype “T Glu a” was more common in HP than in GH or PE (20 versus 6 and 6%, resp.; *P* < 0.0032). Conversely, the haplotype “C Glu a” was more common in GH and PE than in HP (17 and 17 versus 5%; *P* = 0.0061). These findings suggest a contribution of *eNOS* haplotypes to the development of hypertensive disorders of pregnancy (HDP) that is obscured when specific *eNOS* genotypes alone are considered [5].

In addition, we have also examined whether *eNOS* polymorphisms and haplotypes affect the responsiveness to antihypertensive therapy in women with GH or PE [6]. Although we found no significant differences in genotype or allele distributions when responsive and nonresponsive groups were compared (both PE and GH; all *P* > 0.05), *eNOS* haplotype distribution differed in PE (but not in GH) responsive and nonresponsive groups (*P* = 0.0003), thus suggesting that eNOS haplotypes affect the responsiveness to antihypertensive therapy in PE [6].

Once PE is associated with decreased nitric oxide (NO) formation and no previous study had examined whether *eNOS* polymorphisms affect this alteration, we hypothesized that NO bioavailability may be modulated by *eNOS* polymorphisms in pregnancy [7]. No effects in nitrite concentrations were found among PE women with different *eNOS* genotypes and haplotypes (*P* > 0.05). However, the “C Glu b” haplotype, which was more frequent in the HP group than in the PE group (20 versus 5; *P* = 0.0044), was associated with higher nitrite concentrations than the other haplotypes in HP (*P* < 0.05). These findings indicate that *eNOS* polymorphisms affect endogenous NO formation in normal pregnancy, but not in PE and that the “C Glu b” haplotype may protect against the development of PE by increasing endogenous NO formation [7].

We would like to contribute to the *Review Article* of Valenzuela et al. [1] with our data about another candidate gene related with angiogenesis. Abnormal production of matrix metalloproteinases (MMPs), especially MMP-9, may also play a role in HDP [8, 9]. These alterations may result from functional polymorphisms in the promoter region of *MMP-9* gene, which are known to change MMP-9 expression. Therefore, we examined whether the polymorphisms −1562C/T (rs3918242) and −90(CA)_13-25_ (rs2234681) in the promoter of *MMP-9 *were associated with HDP [8, 9]. The CT genotype and T allele for the −1562C/T polymorphism, besides the haplotype including the alleles T and H, were more commonly found in GH, but not in PE, compared with the HP group (both *P* < 0.05), suggesting that *MMP-9* polymorphisms may be associated with GH, but not with PE [8, 9]. In addition, the GH patients with the LH genotype for the −90(CA)_13-25_ polymorphism have higher plasma MMP-9 concentrations than those with other genotypes [8]. Furthermore, we have examined whether MMP-9 polymorphisms affect the responsiveness to antihypertensive therapy in women with GH or PE [8]. The T allele for the −1562C/T polymorphism and the haplotype combining T and H alleles were associated with lack of responsiveness to the antihypertensive therapy in GH, and the haplotype combining C and H alleles was associated with lack of responsiveness to the antihypertensive therapy in PE [8].

Valenzuela et al. [1] have also pointed out that the findings from candidate gene polymorphisms will need to be complemented by evaluation of interaction between genes. We have recently provided an example of epistasis in PE considering the *MMP-9* and *VEGF* polymorphisms [10]. The results from single locus analysis showed significant differences in the distribution of genotypes and alleles for the *VEGF −*634G/C polymorphism when PE was compared to HP and for the *MMP-9 −*1562C/T polymorphism when GH was compared to HP, respectively (all *P* < 0.05). These results are in agreement with our previous findings [2, 9]. However, we have observed a significant interaction between *MMP-9* and *VEGF* genes associated with the PE group compared to HP [10]. The interaction between *MMP-9* and *VEGF* polymorphisms associated with the PE group is obscured when specific genotypes of these single genes are considered, thus highlighting the importance of gene-gene interactions as major determinants to complex diseases, including PE [11].

In conclusion, we consider that the present may contribute to the interesting review article of Valenzuela et al. [1]. The findings and suggestions from our studies on candidate genes to PE may contribute with additional information of clinical relevance to PE susceptibility.
